# Neutrophil Priming in COPD Patients by Bronchitis Visualized by CT-Scans

**Published:** 2017

**Authors:** Leo Koenderman, Adele Lotamloi, Esther Pompe, Firdaus Mohamed Hoesein

**Affiliations:** Department of Respiratory Medicine and Radiology, University Medical Center Utrecht, The Netherlands.

COPD is characterized by at least two phenotypes, a bronchitis type and an emphysematous type, with an important overlap. It is likely that the bronchitis type will benefit more from anti-inflammatory therapy. Unfortunately, it is difficult to differentiate between the two by measurement of lung function, which is routinely used to stage the disease by GOLD criteria. CT scanning can be used to differentiate between the two types of COPD. We have combined analysis of CT images of the lung of COPD patients with systemic inflammation visualized by priming of neutrophil.

Our data indicate that different inflammatory signatures are associated with different phenotypes of COPD. These signatures reflect themselves in differences tissue density measured by CT scanning.

